# Myeloid-specific deletion of autotaxin inhibits rheumatoid arthritis and osteoclastogenesis

**DOI:** 10.3389/fimmu.2024.1481699

**Published:** 2024-12-18

**Authors:** Gwangbeom Heo, Sihyun Jeong, Soyeong Park, Su Jin Kim, Yunna Lee, Seong Ji Woo, Kyunghwan Kim, Byung-Hyun Park, Sang Hoon Rhee, Eunok Im

**Affiliations:** ^1^ Department of Pharmacy, College of Pharmacy, Pusan National University, Busan, Republic of Korea; ^2^ Research Institute for Drug Development, Pusan National University, Busan, Republic of Korea; ^3^ Department of Biochemistry and Molecular Biology, Chonbuk National University Medical School, Jeonju, Republic of Korea; ^4^ Department of Biological Sciences and Biotechnology, Chungbuk National University, Cheongju, Republic of Korea; ^5^ Department of Biological Sciences, Oakland University, Rochester, MI, United States

**Keywords:** rheumatoid arthritis, autotaxin, lipid rafts, osteoclastogenesis, collagen-induced arthritis

## Abstract

Rheumatoid arthritis (RA) is a chronic autoimmune disease characterized by joint swelling, pain, and bone remodeling. We previously reported that autotaxin (ATX) deficiency disrupts lipid rafts in macrophages. Lipid raft disruption results in the dysregulation of RANK signaling, which is crucial for osteoclastogenesis and the pathogenesis of RA. Therefore, we evaluated the effect of ATX deficiency on joint inflammation and osteoclast differentiation. A collagen-induced arthritis mouse model was used with myeloid lineage-restricted Atx-knockout (Atx^ΔME/ΔME^) mice and DBA/1 mice treated with the ATX inhibitor PF-8380. Joint inflammation and bone erosion were visualized using hematoxylin and eosin staining and micro-computed tomography. Osteoclast differentiation was assessed by tartrate-resistant acid phosphatase staining. ATX deficiency suppressed joint inflammation, bone resorption, osteoclast differentiation, and pro-inflammatory protein expression in both Atx^ΔME/ΔME^ mice and PF-8380-treated mice compared to controls. Mean disease score of Atx^+/+^ mice at the end of experiment was 3.813, but that of Atx^ΔME/ΔME^ was 0.185 (p < 0.05). The differentiation of bone marrow-derived macrophages into osteoclasts was reduced in Atx^ΔME/ΔME^ cells compared to Atx^+/+^ cells. ATX deficiency suppressed RANKL-induced phosphorylation of ERK and Akt and the interaction between RANK and TRAF6. ATX deficiency disrupted lipid rafts and dysregulated RANK distribution in RAW264.7 cells. Actin ring formation was also inhibited in Atx^ΔME/ΔME^ osteoclasts. ATX deficiency suppressed RA and osteoclast differentiation by disrupting lipid rafts and altering the RANK signaling pathway. This suggests that ATX inhibition may be an effective strategy for developing new disease-modifying antirheumatic drugs.

## 1 Introduction

Rheumatoid arthritis (RA) is a chronic inflammatory autoimmune disease affecting 0.46% of the global population (1), and its prevalence is gradually increasing in all age groups (2). Strategies for RA therapy include multiple drugs, such as nonsteroidal anti-inflammatory drugs, glucocorticoids, conventional synthetic and biologic disease-modifying antirheumatic drugs (DMARDs) (3, 4). Bone erosion is a key feature for the diagnosis, monitoring, and treatment of RA, and is associated with a poor prognosis (5–7). It is triggered by pro-inflammatory cytokines and receptor activator of nuclear factor kappa B ligand (RANKL) and executed by osteoclasts (8). It is important to stop or delay bone and cartilage erosion because it can cause functional disability in patients with RA.

Osteoclasts are multinucleated giant cells capable of resorbing the bone matrix and differentiating from myeloid precursors in the presence of macrophage colony-stimulating factor (M-CSF) and RANKL (9). Osteoclasts degrade bone matrix by producing enzymes, such as cathepsin K, matrix metalloproteinase 9 (MMP-9), and tartrate-resistant acid phosphatase (TRAP) (10, 11). The signaling cascade of RANKL-regulated osteoclast differentiation begins with RANK. Upon the binding of RANKL to RANK, TRAF6, a key downstream molecule of the RANK signaling pathway, is recruited (12). Afterward, the nuclear factor kappa B (NF-κB) and mitogen-activated protein kinase (MAPK) signaling pathways are triggered (13).

Autotaxin (ATX) is a secretory enzyme that catalyzes the hydrolysis of lysophosphatidylcholine (LPC) to lysophosphatidic acid (LPA) (14). Recent studies have shown that LPA contributes to osteoclast differentiation and arthritis. The expression of ATX and LPA increased in the synovium of patients with RA (15) and increased expression of ATX in primary synovial fibroblasts from mice with arthritis was observed suggesting that LPA induces the proliferation and migration of synovial fibroblasts (16). Osteoclast-specific Atx knockout mice showed reduced bone loss in TNF-α-induced synovitis, lipopolysaccharide (LPS)-induced arthritis, and K/BxN serum transfer-induced arthritis models (17). We recently suggested a novel role for ATX in macrophages. Lipid rafts were disrupted in Atx-deficient primary macrophages and Raw264.7 cells treated with an ATX inhibitor. Disrupted lipid rafts dysregulated the TLR4 signaling pathway in macrophages by disturbing the interaction between TLR4 and its co-receptor cluster of differentiation 14 (18).

TLR2, 3, 4, and 7 are highly expressed in the synovial tissue of patients (19–21). In an animal model of arthritis, a TLR4 antagonist suppressed the clinical manifestation and histological damage of RA (22). The role of TLR4 in osteoclastogenesis is both complex and controversial. Both the inhibitory and pro-survival effects of LPS on osteoclast differentiation have been reported (23). Liu et al. suggested a bifunctional role for TLR4 in osteoclast differentiation. Osteoclastogenesis was inhibited when osteoclast precursors were stimulated with RANKL and LPS simultaneously. However, when bone marrow-derived macrophages (BMMs) were treated with RANKL before LPS treatment, this promoted osteoclastogenesis (24).

Lipid rafts are clusters of lipids, mainly cholesterol and sphingolipids, found on the plasma membrane (25). Lipid rafts act as a signaling and sorting platform for various biochemical reactions within the plasma membrane and have been considered potential therapeutic targets. Ha et al. suggested that lipid rafts play a crucial role in RANKL signaling and osteoclast functioning. They reported that RANK, TRAF6, and Src are recruited to lipid rafts in response to RANKL treatment. They also observed that lipid raft disruption induced by methyl-β-cyclodextrin blocks actin ring formation in osteoclast membranes and hinders osteoclast bone resorption (26, 27). Meanwhile, ATX deficiency disrupts lipid rafts in macrophages, as mentioned above (18). Based on this, we hypothesized that ATX deficiency in macrophages causes RANK dysregulation, which ameliorates arthritis and inhibits osteoclastogenesis. Therefore, we investigated the effects of myeloid-specific ATX deficiency and pharmacological ATX inhibition on joint inflammation and bone destruction in an animal model of arthritis. We also investigated the effect of ATX deficiency on RANKL-induced osteoclastogenesis *in vitro*.

## 2 Materials and methods

### 2.1 Animals

LysM^Cre/+^Atx^loxp/loxp^ (Atx^ΔME/ΔME^) and LysM^+/+^Atx^loxp/loxp^ (Atx^+/+^) mice were kindly provided by Dr. Sang Hoon Rhee (Oakland University, Rochester, MI, USA). Twelve-week-old male Atx^ΔME/ΔME^ and Atx^+/+^ mice, and eleven-week-old male DBA/1 (Orient Bio, Seongnam, Republic of Korea) mice were kept at 20–22°C with a 12 h light–dark cycle. Drinking water and rodent chow (Samtako Bio Korea; Osan, Republic of Korea) were provided *ad libitum*.

### 2.2 Collagen-induced arthritis mouse model

The CIA mouse model was established as described previously (28). Briefly, immunization-grade chicken type II collagen (Chondrex, Redmond, WA, USA) was mixed thoroughly with an equal volume of complete Freund’s adjuvant (CFA) (Chondrex) and the mixture was subcutaneously injected into the mouse tail on day 0. On day 21, collagen was mixed with an equal volume of incomplete Freund’s adjuvant (IFA) (Chondrex) and the uniform mixture was intraperitoneally injected. One day prior to immunization or boosting, 10 mg of immunization grade chicken type II collagen was dissolved in 2.5 ml of 0.05 M acetic acid (Daejung Chemicals and Metals, Siheung, Republic of Korea) diluted in distilled water under gentle shaking at 4°C overnight. To obtain more severe arthritis symptoms, 5 μg of LPS (InvivoGen, San Diego, CA, USA) diluted in 100 μl of normal saline was injected intraperitoneally on day 28 into the DBA/1 mouse. To assess the effects of pharmacological ATX inhibition, the ATX inhibitor PF-8380 (10 mg/kg, diluted in normal saline) was injected intraperitoneally every day from day 30 to the end of the experiment. Hind paw thickness was measured using a caliper. The clinical score was 0–4 for each limb, with a possible total score of 16. The assessment was performed every 2 or 3 days from day 21. Mice were euthanized in a CO_2_ chamber at the end of the experiment. Hind leg tissues were fixed with 10% formalin solution (Sigma-Aldrich, St. Louis, MO, USA) for 1 day at room temperature and stored in phosphate-buffered saline (PBS) (ELPIS-Biotech, Daejeon, Republic of Korea) at 4°C. Ankle and paw tissues were stored in the RNAlater Stabilization Solution (Thermo Fisher Scientific, Waltham, MA, USA) at -80°C.

### 2.3 Micro-computed tomography

Micro-computed tomography (micro-CT) was conducted, and the results were analyzed by the Laboratory Animal Center of Daegu-Gyeongbuk Medical Innovation Foundation (Daegu, Republic of Korea). Radiographs of the knee, ankle, and paw from formalin-fixed hind leg tissues were obtained using Quantum FX micro-CT (Perkin Elmer, Waltham, MA, USA) at 90 kV and 180 μA. Images were analyzed using Analyze 12.0 software (AnalyzeDirect, Overland Park, KS, USA) to obtain total volume (TV, mm^3^), which indicates the volume of bone and tissue of the region of interest (ROI), and bone volume (BV, mm^3^), which indicates the volume of cortical bone and trabecular bone of the ROI. Bone volume fraction (BV/TV) was used as a parameter of bone quality.

### 2.4 Osteoclastogenesis

Osteoclasts were differentiated from BMMs. Bone marrow was obtained from 5- to 7-week-old mice. After euthanasia, the tibia and femur were isolated from the mice. The bones were transferred to Hank’s balanced salt solution (Gibco, Waltham, MA, USA) supplemented with 3% penicillin/streptomycin (P/S). Muscle and connective tissues were removed from bones using lint-free tissue and scissors. Bones were soaked in 70% ethanol (Daejung Chemicals and Metals, Siheung, Republic of Korea) for 2 min and then washed with serum-free α-minimum essential medium (α-MEM) (Welgene, Gyeongsan, Republic of Korea) supplemented with 1% P/S. After cutting both sides of the bone, bone marrow was flushed with serum-free α-MEM supplemented with 1% P/S using a 1-ml syringe and 26G needle until the bone turned into white color. Bone marrow was centrifuged at 250 × *g* for 8 min, and the supernatant was discarded. Next, red blood cells (RBCs) in bone marrow were lysed using RBC lysis buffer for 5 min. The RBC lysis buffer (20 ml, pH 7.3) was prepared from potassium carbonate (13.8 mg, Sigma-Aldrich), ammonium chloride (165.8 mg, Kanto Chemical, Tokyo, Japan), and ethylenediamine tetraacetic acid (EDTA) (0.744 mg, Duchefa Biochemie, Haarlem, Netherlands), and the pH was adjusted with 1 M hydrochloric acid (Daejung Chemicals and Metals). After removal of RBC lysis buffer with centrifugation, cells were resuspended in growth medium; α-MEM supplemented with 10% Fetal bovine serum (FBS) and 1% P/S. Residual debris was removed with cell strainer (SPL Life Sciences, Pocheon, Republic of Korea). Cells were incubated for 16 h in growth medium containing 5 ng/ml of M-CSF (R&D Systems, Minneapolis, MN, USA). Subsequently, non-adherent cells were collected from the supernatant, and these cells were considered as bone marrow-derived monocytes. The bone marrow-derived monocytes were plated for each experiment and further cultured for 3 days in growth medium containing 30 ng/ml of M-CSF. The resulting cells were considered as BMMs. BMMs were stimulated with 100 ng/ml of soluble RANKL (sRANKL) (Peprotech, Rocky Hill, NJ, USA) and 30 ng/ml of M-CSF in growth medium. The cells were then incubated for additional days depending on each experiment scheme.

### 2.5 TRAP staining

Cells were fixed with 10% formalin solution (Sigma-Aldrich) at room temperature for 10 min. The cells were permeabilized with 0.05% Triton X-100 (Daejung Chemicals and Metals) at room temperature for 5 min. Next, the cells were stained with TRAP staining solution at 37°C for 20 min in darkness. The cells were washed with PBS between each step. TRAP staining solution was freshly prepared from basic incubation medium, naphthol mix, and fast red violet (Sigma-Aldrich). For example, to prepare 2 ml of TRAP staining solution, 2 ml of basic incubation medium, 10 μl of naphthol mix, and 1.2 mg of fast red violet were mixed. For 200 ml of basic incubation medium (pH 4.7–5.0), 1.84 g of sodium acetate anhydrous (Sigma-Aldrich), 2.28 g of L-tartrate dibasic dihydrate (Sigma-Aldrich), and 560 μl of glacial acetic acid (Junsei Chemical, Tokyo, Japan) were dissolved in distilled water, and the pH was adjusted with 5 M sodium hydroxide (Sigma-Aldrich). For 100 μl of naphthol mix, 2 mg of naphthol AS-MX phosphate (Sigma-Aldrich) was dissolved in 100 μl of ethoxyethanol (Sigma-Aldrich). Cells were visualized under brightfield microscopy (Nikon, Tokyo, Japan). For histology, formalin-fixed paraffin-embedded tissues on glass slides were deparaffinized and rehydrated with xylene (Duksan Pure Chemicals, Ansan, Republic of Korea) and serial ethanol dilution. Deparaffinized tissue was stained with TRAP staining solution with the same procedure as cell staining. After staining, the tissue was dehydrated with serial dilution of ethanol and xylene.

### 2.6 Immunohistochemistry

Hind leg tissue was decalcified in 10% EDTA (Duchefa Biochemie, Haarlem, Netherlands) solution for 4 weeks. EDTA solution was renewed every week. Paraffin embedding and hematoxylin and eosin (H&E) staining were performed by LOGONE Bio-Convergence Research Foundation (Seoul, Republic of Korea) and Imaging Core Laboratory of Pusan National University (Yangsan, Republic of Korea). Safranin O staining was performed by CM Biopath (Hwaseong, Republic of Korea).

### 2.7 Immunoblotting analysis

Cells were harvested using a cell scraper. Tissues were frozen in liquid nitrogen and then homogenized with mortar and pestle. Total proteins from cells and tissues were extracted with protein extraction solution (ELPIS-Biotech, Daejeon, Republic of Korea) supplemented with protease inhibitors (Roche, Basel, Switzerland) and phosphatase inhibitors (Sigma-Aldrich). The protein concentration was determined using a Pierce BCA Protein Assay Kit (Thermo Fisher Scientific, Waltham, MA, USA) following the manufacturer’s instruction. Equal amounts of proteins for each group were separated with sodium dodecyl sulfate (SDS)-polyacrylamide gel electrophoresis. Proteins on the gel were transferred to polyvinylidene difluoride membrane (Merck, Kenilworth, NJ, USA). The membranes were blocked with skim milk (BD Biosciences, Franklin Lakes, NJ, USA) or bovine serum albumin (MP Biomedicals, Santa Ana, CA, USA) and then incubated in primary antibody at 4°C overnight. The membranes were incubated with secondary antibody adequate for each primary antibody at room temperature for 1 h. Antibodies against p-ERK1/2, ERK1/2, p-Akt, Akt, p-STAT3, STAT3, NF-κB p65, RANK, and Flotillin-1 were purchased from Cell Signaling Technology (Danvers, MA, USA). Antibodies against β-actin and TRAF6 were purchased from Sigma-Aldrich and MBL (Tokyo, Japan), respectively. Secondary antibodies were purchased from Enzo Life Sciences (Farmingdale, NY, USA). Proteins were visualized using an enhanced chemiluminescence reagent (Advansta, San Jose, CA, USA) and the ChemiDoc Gel Imaging System (Bio-Rad Laboratories, Hercules, CA, USA).

### 2.8 Immunoprecipitation

Total protein extracts were prepared with protein extraction buffer supplemented with protease inhibitor. Equal amounts of proteins for each group were incubated with primary antibody at 4°C overnight. Protein G PLUS-agarose bead (Santa Cruz Biotechnology, Dallas, TX, USA) was blocked with 1% BSA in protein extraction buffer at 4°C for 1 h. Then the bead was added to primary antibody-treated protein extracts and incubated at 4°C for 4 h. Bead was washed with PBS and incubated with protein sample buffer containing 5% SDS (ELPIS-Biotech) at 95°C for 5 min. Eluted samples were analyzed by immunoblotting analysis.

### 2.9 Enzyme-linked immunosorbent assay

Total proteins were extracted from ankle and paw tissues with the same method as used in immunoblotting analysis. TNF-α concentration in protein lysates was determined by ELISA following the manufacturer’s instruction (BioLegend, San Diego, CA, USA). The measured value was normalized to the total protein concentration of each sample.

### 2.10 Reverse transcription polymerase chain reaction

Cells were harvested using a scraper. Tissues were frozen with liquid nitrogen and then homogenized with mortar and pestle. Total RNA from ankle tissue or cells was isolated with RiboEx reagent (GeneAll Biotechnology, Seoul, Republic of Korea). The concentration and purity of the total RNA were measured with μDrop™ Plate and spectrophotometer (Thermo Fisher Scientific). One microgram of the total RNA was used for complementary DNA (cDNA) synthesis. cDNA was synthesized using RT-&GO™ reagent (MP Biomedicals) and oligo dT primer (ELPIS-Biotech) following the manufacturers’ instruction. Maxime™ PCR PreMix Kit (i-StarTaq) (iNtRON Biotechnology, Seongnam, Republic of Korea) was used for RT-PCR following the manufacturer’s instruction. All primer sequences are presented in **Table 1**.

**Table 1:** Primer sequences for RT-PCR analysis.

Gene	Sequence	
Mouse Mmp9	Sense	5′-CTGTCCAGACCAAGGGTACAGCCT-3′
	Antisense	5′-GTGGTATAGTGGGACACATAGTGG-3′
Mouse Mmp13	Sense	5′-CCAGAACTTCCCAACCATGT-3′
	Antisense	5′-GTCTTCCCCGTGTTCTCAAA-3′
Mouse Cathepsin *K*	Sense	5′-AGGCGGCTATATGACCACTG-3′
	Antisense	5′-CCGAGCCAAGAGAGCATATC-3′
Mouse Trap	Sense	5′-ACACAGTGATGCTGTGTGGCAACTC-3′
	Antisense	5′-CCAGAGGCTTCCACATATATGATGG-3′
Mouse Gapdh	Sense	5′-CTCACTGGCATGGCCTTCCG-3′
	Antisense	5′-ACCACCCTGTTGCTGTAGCC-3′

### 2.11 Cell culture

Raw264.7, a mouse macrophage cell line, was purchased from American Type Culture Collection (Manassas, VA, USA). Raw264.7 cells were cultured in Dulbecco’s Modified Eagle’s Medium (Hyclone, Logan, UT, USA). All cells were incubated at 37°C in a humidified 5% CO_2_ incubator and all culture media were supplemented with 10% FBS (Hyclone) and 1% P/S (Hyclone).

### 2.12 Cell viability assay

Raw264.7 cells were plated at a density of 5.0 × 10^3^ cells/well in a 96-well plate. The cells were incubated for 24 h and then treated with vehicle 0.1% dimethyl sulfoxide (Duchefa Biochemie) or 50 μM of PF-8380 (Selleck Chemicals, Houston, TX, USA) in sextuplicate for indicated time. Ten-fold 3-(4,5-dimethyl-2-thiazolyl)-2,5-diphenyl-2H-tetrazolium bromide (MTT) (Sigma-Aldrich) was dissolved in PBS and further diluted in culture medium at a working concentration of 0.5 mg/ml. The cells were incubated in culture medium containing MTT solution for 2 h in darkness. Next, the culture medium was aspirated, and dimethyl sulfoxide was added to dissolve the formazan crystals. The absorbance was measured using a microplate spectrophotometer (Thermo Fisher Scientific).

### 2.13 Bone resorption assay

Bovine bone slices were purchased from Immunodiagnostic Systems (East Boldon, UK). Bone slices were placed in 96-well plate. Bone marrow-derived monocytes were plated on the bone slices and incubated in α-MEM supplemented with M-CSF (30 ng/ml) for 3 days. Then the cells were further incubated with M-CSF (30 ng/ml) and RANKL (100 ng/ml) for 5 more days. Cells were removed from bone slices with 0.2% Triton X-100 in 1M NaCl for 20 min at room temperature. After washing with distilled water, the bone slices stained with hematoxylin. Stained bone slices were observed with brightfield microscopy. Resorbed area was analyzed with ImageJ software (NIH, Bethesda, MD, USA).

### 2.14 Cell fractionation

TNE buffer was prepared from 25 mM Tris-HCl (Duchefa Biochemie, Haarlem, Netherlands), 150 mM NaCl (VWR International, Radnor, PA, USA), and 5 mM EDTA. The cells were harvested using a cell scraper. Cells were lysed with cold 1% Triton X-100 in TNE buffer supplemented with protease inhibitors for 30 min on ice. The lysates were centrifuged at 21,000 × *g* for 10 min. The supernatant was obtained and considered to be a non-lipid raft fraction and named the soluble fraction. The residual pellet was washed with 1% Triton X-100 in TNE buffer and centrifuged again at 21,000 × *g* for 5 min. The cells were washed twice. Next, the pellet was lysed with a protein extraction solution containing SDS and supplemented with protease inhibitors for 1 h on ice. The lysates were centrifuged at 21,000 × *g* for 10 min. The supernatant was obtained and considered the lipid raft fraction and named the insoluble fraction. Protein expression was analyzed by immunoblotting.

### 2.15 Actin filament staining

The cells were fixed and permeabilized as described in Section 2.7. Cells were washed with PBS and stained with Phalloidin-iFluor 647 Reagent (ab176759, Abcam, Cambridge, United Kingdom) in 1% BSA in PBS at room temperature for 1 h. Cells were then washed with PBS and sealed with VECTASHIELD^®^ Antifade Mounting Medium with DAPI (Vector Laboratories, Burlingame, CA, USA). The cells were observed under a confocal microscope (LSM900, ZEISS, Oberkochen, Germany).

### 2.16 Immunofluorescence

Hind leg tissue slides were deparaffinized and rehydrated by xylene and serial dilution of ethanol. Antigen retrieval was performed by heat-induced epitope retrieval method. The slides were incubated in blocking buffer (X0909, Dako, CA, USA). Then the slides were incubated with CD31 (#3266925, Sigma-Aldrich) and F4/80 antibody (sc-377009, Santa Cruz Biotechnology) at 4°C overnight. Slides were washed with TBS-T several times and then incubated again with the FITC-conjugated secondary antibody (A120-101F, Bethyl Laboratories, Inc.). Before mounting, slides were counterstained with DAPI solution (H-1200, Vector Laboratories). Fluorescence images were obtained using Leica DMi8 (Leica, Wetzlar, German) at 200× and 400× magnification.

### 2.17 Statistical analysis

Statistical analysis was performed using GraphPad Prism (Version 5.03; GraphPad Software, San Diego, CA, USA). Data are presented as mean ± standard error of the mean. Differences between groups were determined by *t*-test or Mann-Whitney test or one-way or two-way analysis of variance, followed by Tukey’s and Bonferroni’s *post-hoc* tests.

### 2.18 Ethics approval

All procedures involving animals were reviewed and approved by the Pusan National University Institutional Animal Care and Use Committee (No. 2021-2876, Busan, Republic of Korea).

## 3 Results

### 3.1 Arthritis symptoms in myeloid lineage-restricted Atx knockout mice

**Figure 1:**
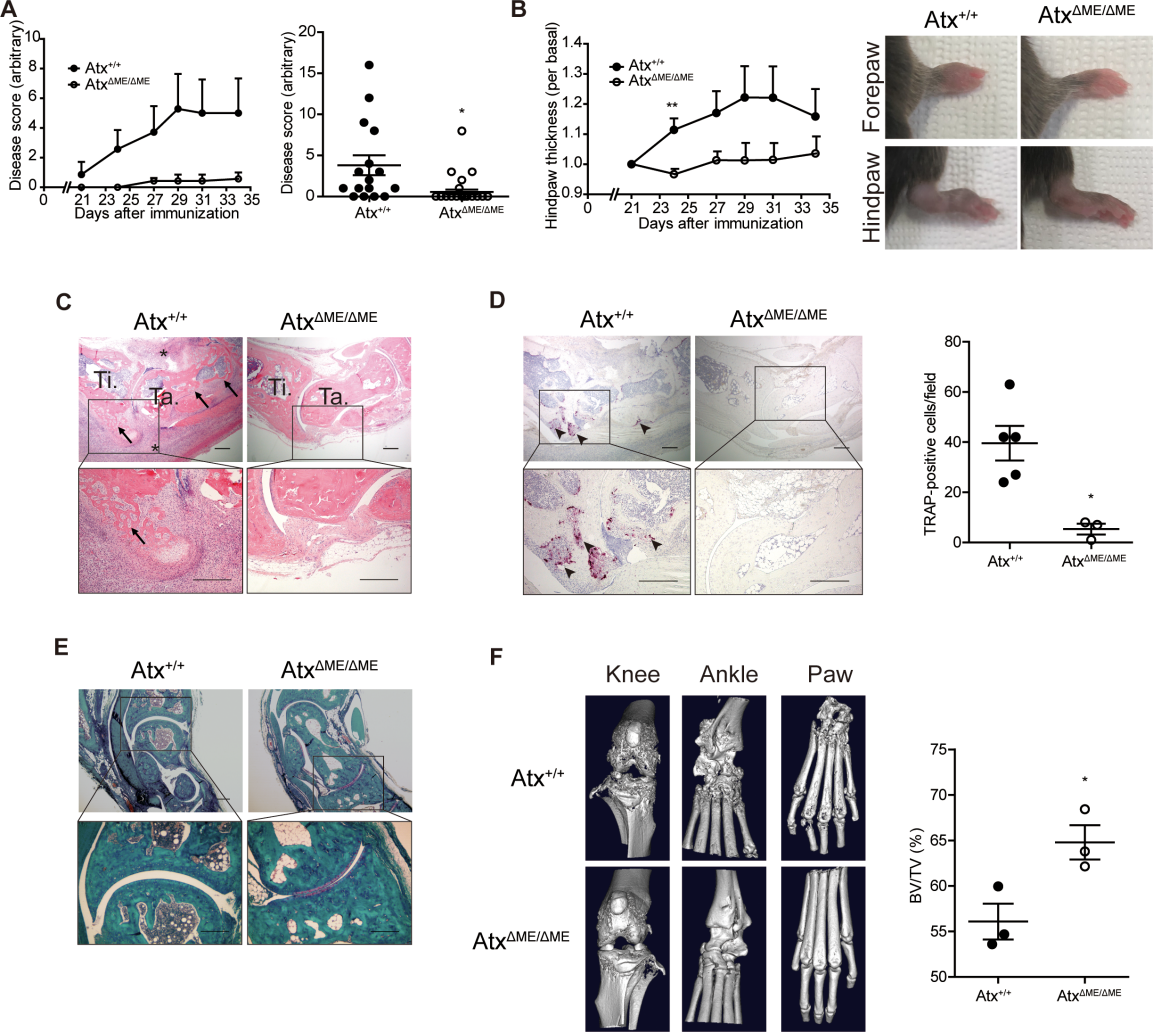
Myeloid-specific deletion of Atx ameliorated arthritis in a collagen-induced arthritis (CIA) model. CIA models were established using Atx^+/+^ and Atx^ΔME/ΔME^ mice. **(A)** The disease score was assessed on a 0–4 scale for each limb. Disease scores measured every other day after immunization (left panel) and at the end point of experiments (right panel, n = 16 for Atx^+/+^ mice, n = 17 for Atx^ΔME/ΔME^ mice) were shown. **(B)** Hind paw thickness was measured using a caliper (left panel). Representative images of the paws at the end of experiment (right panel). **(C)** Representative H&E staining images of ankle tissues. Arrows indicate bone erosion. Asterisks indicate immune cell infiltration. **(D)** Representative images of TRAP staining images (left panel). Arrow heads indicate TRAP-positive cells. TRAP-positive cells were counted for each visual field (right panel). **(E)** Representative Safranin O staining images of ankle tissues. **(F)** Representative micro-CT images of the knee, ankle, and hind paw (left panel). Morphometric analysis quantified bone destruction in the metatarsals and phalanges (right panel). Scale bar = 200 μm. *p < 0.05 and **p < 0.01. TRAP, tartrate-resistant acid phosphatase; BV/TV, bone volume/total volume.

A previous study has reported that ATX and LPA inhibition using chemical antagonists suppress osteoclast differentiation, and osteoclast-specific deletion of Atx ameliorates arthritis in animal models (17). Considering these previous findings and the fact that osteoclasts differentiate from myeloid precursors, we investigated the effects of myeloid-specific ATX deficiency on osteoclastogenesis and synovial inflammation in an animal model of arthritis. In this study, we used myeloid lineage-restricted Atx-knockout (Atx^ΔME/ΔME^) mice and a CIA mouse model. Atx^ΔME/ΔME^ mice showed a lower disease score and incidence compared to Atx^+/+^ mice. Twelve of 16 Atx^+/+^ mice and five of 17 Atx^ΔME/ΔME^ mice exhibited disease symptoms at the end of the experiment (**Figure 1A**). Consistently, hind paw swelling was reduced in Atx^ΔME/ΔME^ mice compared to that in Atx^+/+^mice (**Figure 1B**). Bone erosion and inflammatory cell infiltration in the synovial tissue were reduced in Atx^ΔME/ΔME^ mice compared to those in Atx^+/+^ mice (**Figure 1C**). Osteoclasts were visualized by TRAP staining. There were significantly fewer TRAP-positive cells in the hind leg tissues of Atx^ΔME/ΔME^ mice compared to those in Atx^+/+^ mice (**Figure 1D**). Reduction in cartilage loss was also observed in Atx^ΔME/ΔME^ mice (**Figure 1E**). Bone destruction in hind leg tissue was assessed using micro-CT analysis. Consistent with the histological damage shown in H&E staining images, micro-CT images showed that bone destruction in the knee, ankle, and paw was ameliorated in Atx^ΔME/ΔME^ mice compared to that in Atx^+/+^ mice (**Figure 1F**). Morphometric analysis of the metatarsals and phalanges showed the suppressive effects of myeloid-specific Atx deletion on bone erosion. The bone volume (BV) to total volume (TV) ratio was significantly increased in Atx^ΔME/ΔME^ mice compared to that in Atx^+/+^ mice, indicating that myeloid-specific ATX deficiency inhibited bone loss in a CIA mouse model. These results demonstrate that myeloid lineage-restricted ATX deficiency reduces joint inflammation and bone erosion in a CIA mouse model.

### 3.2 CIA development in ATX inhibitor-treated mice

**Figure 2:**
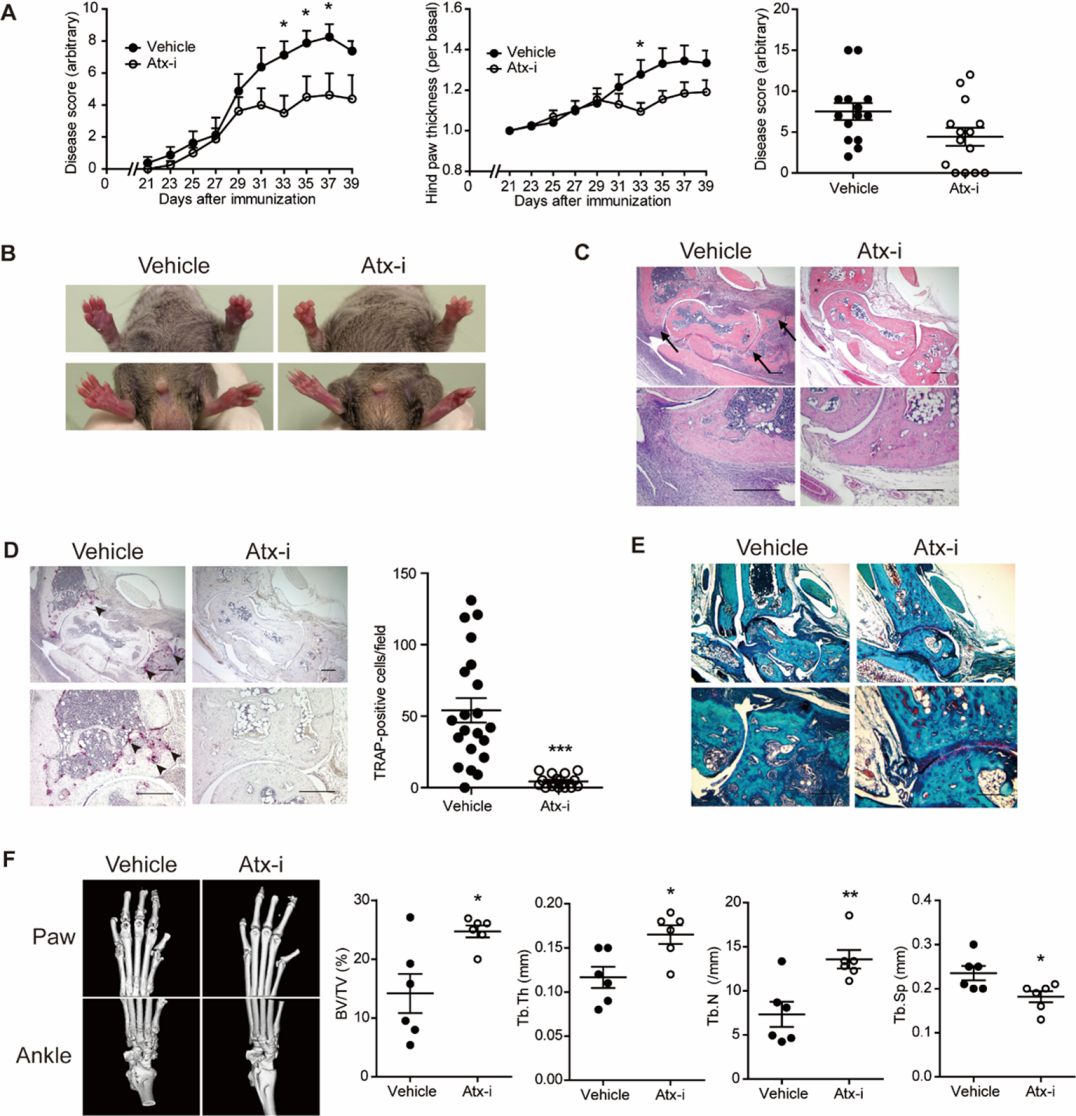
The ATX inhibitor PF-8380 ameliorated CIA. CIA models were established using DBA/1 mice and the ATX inhibitor PF-8380 (10 mg/kg). **(A)** The disease score was assessed on a 0–4 scale for each limb (left panel). Disease scores at the end point of two independent experiments (right panel, n = 14). **(B)** Hind paw thickness was measured using a caliper (left panel). Representative images of the paws at the end of the experiment (right panel). **(C)** Representative H&E staining images of ankle tissues. Arrows indicate bone erosion. **(D)** Representative images of TRAP staining images (left panel). Arrow heads indicate TRAP-positive cells. TRAP-positive cells were counted for each visual field (right panel, n = 21 for Veh group, n = 17 for Atx-i group). **(E)** Representative Safranin O staining images of ankle tissues. **(F)** Representative micro-CT images of the hind paw and ankle (left panel). Morphometric analysis quantified bone destruction in the metatarsals and phalanges (right panel). Scale bar = 200 μm. *p < 0.05, **p < 0.005 and ***p < 0.001. Atx-i, ATX inhibitor; TRAP, tartrate-resistant acid phosphatase; BV/TV, bone volume/total volume.

Due to the relatively low incidence and mild symptoms of arthritis in the C57BL/6 strain, additional investigations were conducted using DBA/1 mice, which are highly susceptible to CIA (29). PF-8380, a potent orthosteric inhibitor of ATX, was used to investigate the efficacy of the pharmacological inhibition of ATX in an arthritis mouse model (30, 31). Consistent with the results in Atx^ΔME/ΔME^ mice (**Figure 1**), pharmacological inhibition of ATX ameliorated the arthritis symptoms in DBA/1 mice (**Figure 2A**). Hind paw swelling was also reduced in ATX inhibitor-treated mice compared to that in vehicle-treated mice (**Figure 2B**). H&E staining images of ankle tissues showed that bone erosion in the tibia and talus was suppressed in ATX inhibitor-treated mice compared to that in vehicle-treated mice. Inflammatory infiltration was also reduced in ATX inhibitor-treated mice compared to that in vehicle-treated mice (**Figure 2C**). The number of TRAP-positive cells was reduced in ATX inhibitor-treated mice compared to that in vehicle-treated mice (**Figure 2D**). Cartilage damage was found to be decreased in ATX inhibitor-treated mice as well (**Figure 2E**). Bone erosion was further investigated using micro-CT analysis (**Figure 2F**). Representative images show that bone erosion in the metatarsals and phalanges was reduced in ATX inhibitor-treated mice compared to that in vehicle-treated mice. Morphometric analysis of the metatarsals and phalanges indicated relief effects of the ATX inhibitor on bone erosion. The bone volume-to-total volume ratio was significantly increased in ATX inhibitor-treated mice compared to that in vehicle-treated mice. These results suggest that the pharmacological inhibition of ATX suppresses joint inflammation and bone erosion in a CIA mouse model.

### 3.3 Suppressed expression of inflammatory proteins and osteoclastogenic genes in ATX inhibitory states

**Figure 3:**
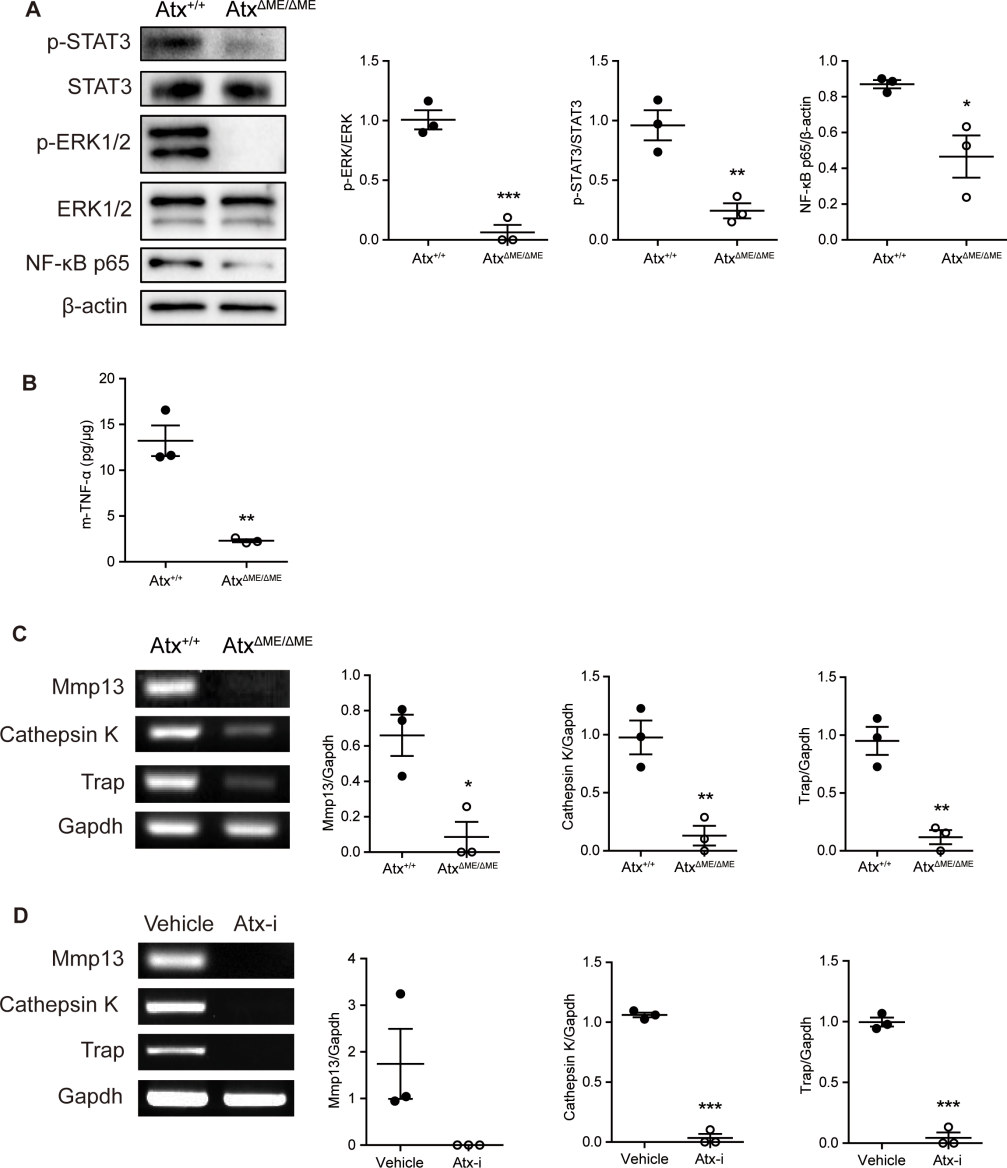
ATX deficiency suppressed the expression levels of inflammatory-related proteins and osteoclast-related genes in a CIA model. Proteins and total RNA were extracted from ankle tissues. **(A)** The expression of STAT3, ERK1/2, and NF-κB p65 was examined by immunoblotting analysis. **(B)** TNF-α level in ankle tissue was analyzed by ELISA. **(C, D)** The expression of Mmp9, Mmp13, Cathepsin K, and Trap in ankle tissue was examined by RT-PCR. *p < 0.05, **p < 0.005 and ***p < 0.001. STAT3, signal transducer and activator of transcription 3; ERK1/2, extracellular signal-regulated kinase1/2; NF-κB, nuclear factor kappa B; m-TNF-α, mouse tumor necrosis factor alpha; Mmp9, matrix metallopeptidase 9; Mmp13, matrix metallopeptidase 13; Trap, tartrate-resistant acid phosphatase; Gapdh, glyceraldehyde 3-phosphate dehydrogenase; Atx-i, ATX inhibitor.

The expression of proteins associated with joint inflammation was investigated. The phosphorylation of STAT3 is upregulated under inflammatory conditions and induces inflammatory cytokine and RANKL expression (32). The level of p-STAT3 was suppressed in Atx^ΔME/ΔME^ mice compared to that in Atx^+/+^ mice (**Figure 3A**). Phosphorylation of extracellular signal-regulated kinase (33), a key mediator of osteoclast differentiation (34, 35), was also reduced in Atx^ΔME/ΔME^ mice compared to that in Atx^+/+^ mice (**Figure 3A**). The expression of NF-κB p65, a well-known pro-inflammatory transcription factor (35), was reduced in Atx^ΔME/ΔME^ mice compared to that in Atx^+/+^ mice (**Figure 3A**). As shown in **Figure 3B**, TNF-α expression in Atx^ΔME/ΔME^ mice was lower than that in Atx^+/+^ mice. In addition to inflammatory protein expression, the mRNA expression of osteoclast-related genes was also investigated. Cathepsin K is an enzyme that degrades collagen in joints (36). TRAP is responsible for bone resorption and is widely used as an osteoclastic marker (37). MMPs are highly involved in RA by degrading cartilage and inducing joint tissue destruction (38). As shown in **Figure 3C**, the expression levels of Mmp9, Mmp13, Cathepsin K and Trap were decreased in Atx^ΔME/ΔME^ mice compared to those in Atx^+/+^ mice. Similarly, the expression levels of these genes in ATX inhibitor-treated mice were lower than those in vehicle-treated mice (**Figure 3D**). These results suggest that ATX deficiency blocks not only inflammatory responses but also osteoclast differentiation in a CIA mouse model.

### 3.4 Suppression of osteoclastogenesis by ATX deficiency and LPS treatment

**Figure 4:**
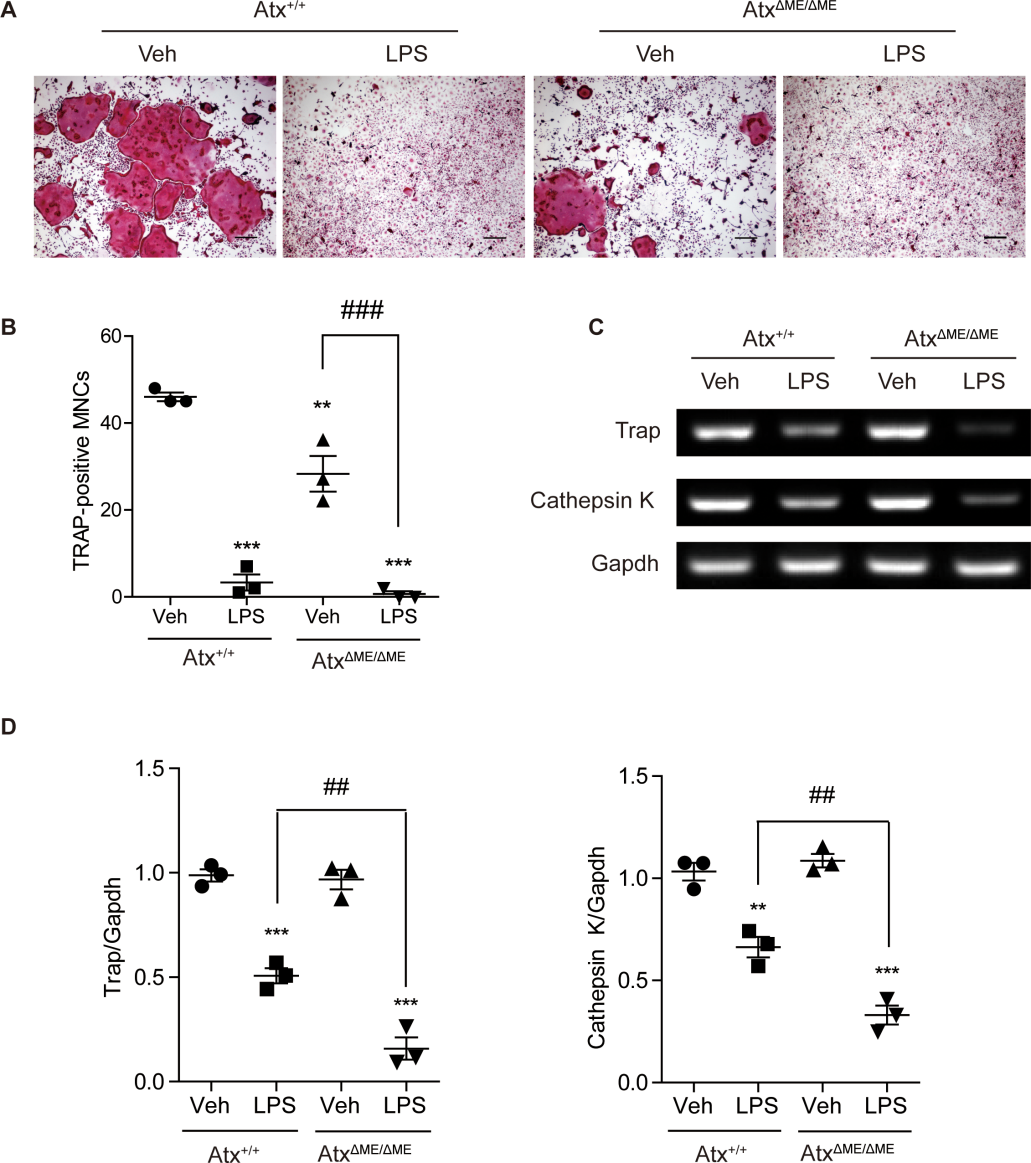
Effects of ATX deficiency and LPS on osteoclastogenesis of bone marrow-derived macrophages (BMMs) were assessed with TRAP staining, and osteoclast-related gene expression was analyzed by RT-PCR. **(A)** BMMs were incubated with M-CSF (30 ng/ml) and RANKL (100 ng/ml) in the presence or absence of LPS (5 ng/ml) for 4 days. TRAP-stained cells were visualized under the brightfield microscope. **(B)** TRAP-positive multinucleated cells with more than three nuclei were counted. **(C)** BMMs were incubated with M-CSF (30 ng/ml) and RANKL (100 ng/ml) in the presence or absence of LPS (5 ng/ml) for 5 days. Total RNA was isolated from the cells, and mRNA expression was analyzed by RT-PCR. **(D)** Each gene expression was normalized to GAPDH expression. **p < 0.01 and ***p < 0.001 compared to the vehicle-treated Atx^+/+^ cells. ^##^p < 0.01 and ^###^p < 0.001. **p < 0.005 and ***p < 0.001. Scale bar=200 μm. Veh, vehicle; LPS, lipopolysaccharide; MNCs, multinucleated cells; Trap, tartrate-resistant acid phosphatase; Gapdh, glyceraldehyde 3-phosphate dehydrogenase.

To investigate the effects of ATX deficiency on osteoclastogenesis *in vitro*, osteoclastogenesis in BMMs was examined. Representative images of TRAP-stained cells showed that Atx^ΔME/ΔME^ BMMs were less differentiated into osteoclasts than Atx^+/+^ BMMs (**Figure 4A**). The number of TRAP-positive multinucleated cells is presented in **Figure 4B**, indicating that Atx^ΔME/ΔME^ BMMs were significantly less differentiated into osteoclasts than Atx^+/+^ BMMs. These results confirmed the suppressive effect of ATX deficiency on osteoclastogenesis.

As mentioned above, we previously reported that ATX deficiency causes lipid raft disruption, which abolishes LPS-induced TLR4 signaling (18). Another group suggested that simultaneous treatment with LPS and RANKL suppresses osteoclastogenesis (24). Based on previous studies, the effects of ATX deficiency on osteoclast differentiation were investigated in the presence of LPS. LPS-treated Atx^+/+^ BMMs showed a lower degree of osteoclastogenesis than vehicle-treated Atx^+/+^ BMMs (**Figures 4A, B**). Intriguingly, LPS-treated Atx^ΔME/ΔME^ BMMs also showed a lower degree of osteoclastogenesis than vehicle-treated Atx^ΔME/ΔME^ BMMs. LPS-treated Atx^ΔME/ΔME^ BMMs showed a slightly lower degree of osteoclastogenesis than LPS-treated Atx^+/+^ BMMs. Furthermore, LPS treatment inhibited the expression of osteoclast-related genes, such as Trap and cathepsin K, in both Atx^+/+^ BMMs and Atx^ΔME/ΔME^ BMMs (**Figure 4C, D**). These results suggest that LPS inhibits osteoclastogenesis via an ATX-independent pathway.

### 3.5 Dysregulated lipid raft integrity and osteoclastogenic signaling in ATX inhibitory states

**Figure 5:**
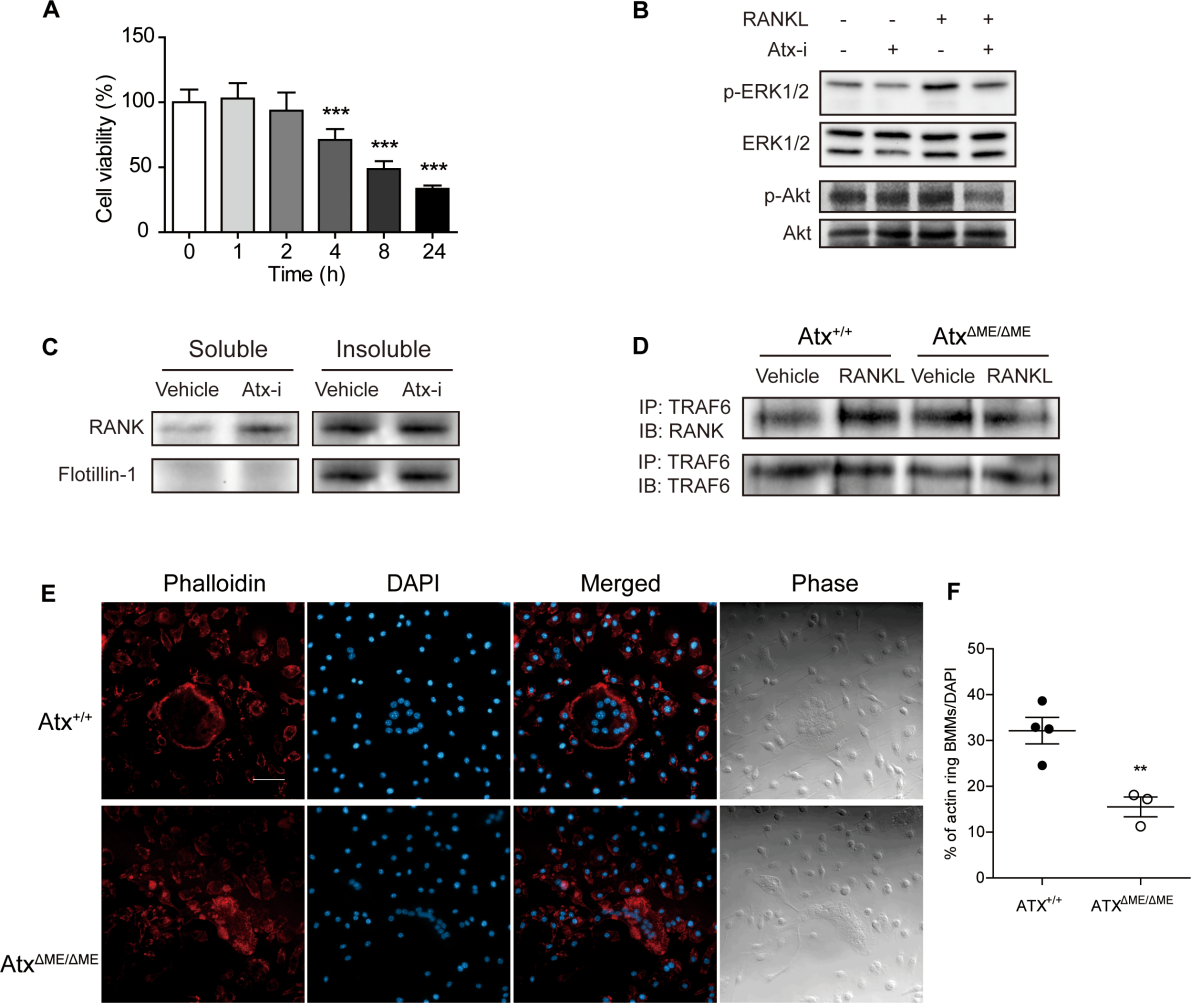
ATX deficiency suppressed RANK signaling and dysregulated actin ring formation by disrupting lipid rafts. **(A)** Effects of ATX inhibitor PF-8380 on Raw264.7 cell proliferation was investigated by MTT assay. Raw264.7 cells were treated with ATX inhibitor at a concentration of 50 μM for indicated time. **(B)** Raw264.7 cells were treated with 50 μM ATX inhibitor for 1 h and further treated with 100 ng/ml RANKL for 15 min. Protein expression wasmeasured with immunoblotting analysis. **(C)** Lipid rafts were fractionated using Triton X-100. Raw264.7 cells were treated with vehicle or 50 μM ATX inhibitor for 75 min. The soluble to non-ionic detergent fraction was obtained using lysis buffer with Triton X-100. The insoluble to non-ionic detergent fraction was obtained using lysis buffer with SDS. Protein expression was measured with immunoblotting analysis. **(D)** Bone marrow-derived macrophages (BMMs) were incubated with RANKL (100 ng/ml) or vehicle for 15 min. Cell lysates were co-immunoprecipitated with RANK and TRAF6. **(E)** BMMs were incubated with M-CSF (30 ng/ml) and RANKL (100 ng/ml) for 5 days. Actin filaments were stained with phalloidin. **(F)** The ratio of the cells with actin filaments to total cells per slide was presented. **p < 0.01 and ***p < 0.001 compared to the vehicle-treated cells. Scale bar = 50 μm. Atx-i, ATX inhibitor; ERK1/2, extracellular signal-regulated kinase1/2; RANKL, receptor activator of nuclear factor kappa B ligand; RANK, receptor activator of nuclear factor kappa B; TRAF6, tumor necrosis factor receptor-associated factor 6; DAPI, 4′,6–diamidino–2–phenylindole.

Considering that the ATX inhibitor PF-8380 significantly inhibited inflammatory responses in DBA/1 mice with CIA, the effect of the ATX inhibitor was further confirmed *in vitro* using Raw264.7 cells, a mouse macrophage cell line. Prior to immunoblotting analysis, the effects of the ATX inhibitor on cell viability were investigated using an MTT assay to determine the non-cytotoxic concentration and treatment time (**Figure 5A**). Treatment with the ATX inhibitor at a concentration of 50 μM for 4 h exhibited an anti-proliferative effect on Raw264.7 cells. For immunoblotting analysis, cells were treated with vehicle or the ATX inhibitor (50 μM) for 1 h and then treated with vehicle or RANKL (100 ng/ml) for 15 min. As shown in **Figures 5B**, the ATX inhibitor suppressed RANKL-induced phosphorylation of ERK1/2 and Aktin Raw264.7 cells.

Previous reports have suggested that lipid rafts are important for RANK signaling, and ATX alters lipid raft structure; therefore, non-ionic detergent-mediated lipid raft fractionation was conducted to investigate the role of ATX in lipid raft integrity (18, 27). Raw264.7 cells were treated with vehicle or the ATX inhibitor (50 μM) for 75 min. As shown in **Figure 5C**, flotillin-1, a marker of lipid rafts, was localized in the insoluble fraction of the vehicle-treated cells. The flotillin-1 localization was reduced in ATX inhibitor-treated cells compared to that in vehicle-treated cells. Moreover, RANK distribution was dysregulated by ATX inhibition. In the soluble fraction, RANK expression in ATX inhibitor-treated cells was higher than that in vehicle-treated cells. Dysregulation of RANK signaling was further investigated using a co-immunoprecipitation assay. BMMs were treated with RANKL (100 ng/ml) for 15 min and total lysates were isolated. As shown in **Figure 5D**, RANKL-induced binding between RANK and TRAF6 was reduced in Atx^ΔME/ΔME^ BMMs compared to that in Atx^+/+^ BMMs.

Since the actin-based resorption complex is essential for bone resorbing activity and lipid raft disruption blocks actin ring formation, actin ring formation in ATX-deficient cells was investigated with phalloidin staining (26, 39). BMMs were cultured with M-CSF (30 ng/ml) and RANKL (100 ng/ml) for 5 days. The cells were fixed and stained with phalloidin. As shown in **Figure 5E**, Atx^+/+^ cells showed a well-structured actin ring along the border of osteoclasts. In contrast, Atx^ΔME/ΔME^ cells did not exhibit an actin ring and displayed a dispersed staining pattern. These results suggest that ATX inhibition disrupts the structure of lipid rafts, thereby impeding RANK signaling.

## 4 Discussion

This study revealed that articular inflammation and bone erosion were ameliorated by myeloid lineage-restricted deletion of Atx and via pharmacological inhibition of ATX with PF-8380 in a CIA mouse model. ATX deficiency inhibited osteoclastogenesis in the ankle tissues of mice with CIA, thereby hindering bone destruction in the ATX-deficient groups compared to that in the normal groups. The anti-osteoclastogenic effects of ATX deficiency were further confirmed by *in vitro* osteoclast formation experiments using BMMs. RANK-TRAF6 interaction and ERK and Akt phosphorylation were suppressed under ATX-deficient conditions. In contrast, the anti-inflammatory effects of ATX deficiency were demonstrated in our previous report. We had previously reported that macrophage function is hindered by ATX deficiency (18). Phagocytosis and TLR4 signaling were hindered in ATX-deficient macrophages. Since TLR4-induced cytokine production by macrophages was significantly increased in patients with RA, defective TLR4 signaling in ATX-deficient macrophages may be a reason for the anti-inflammatory effect of ATX deficiency in a CIA mouse model (40). We confirmed that ATX deficiency suppresses the expression of NF-κB and TNF-α in a CIA mouse model.

Although most studies regarding ATX have focused on LPC and LPA, this study concentrated on another subject to elucidate the underlying mechanism of the anti-inflammatory and anti-osteoclastogenic effects of ATX deficiency. Our previous study revealed that ATX deficiency induces lipid raft disruption, thereby causing dysregulation of the TLR4 signaling pathway in primary macrophages and Raw264.7 cells (18). Lipid rafts are essential for osteoclast differentiation as they provide RANKL, RANK, TRAF6, and Src with a platform to form a signaling complex (26, 27, 41). Based on these previous findings, we assumed that the anti-osteoclastogenic effects of ATX deficiency may result from lipid raft disruption, which induces dysregulation of the RANK signaling pathway. By using Raw264.7 cells an ATX inhibitor, we confirmed that ATX inhibition disrupted lipid rafts. In addition, ATX inhibition shifted RANK localization from the lipid raft fraction to the non-raft fraction. As RANK localization in lipid rafts is crucial for RANK signaling, the increase in RANK localization in the non-raft fraction induced by ATX inhibition implies a reduction in RANK signaling and suppression of osteoclast differentiation (26).

Previously, Flammier et al. thoroughly investigated the role of ATX in inflammatory bone loss (17). Our findings are consistent with their findings that ATX inhibition is protective against arthritis. The major difference between the previous study and our current study is that their model involved mature osteoclast-specific Atx deficiency, while our model employed myeloid lineage-specific Atx deficiency. In our model, osteoclast precursors lack Atx, allowing us to explore the role of Atx in the osteoclastogenesis. We observed reduced osteoclast differentiation and activity in cells from Atx^ΔME/ΔME^ mice, compared to cells from Atx^+/+^ mice. On the other hand, Flammier et al. showed no difference in bone mass and number of osteoclasts between control mice and osteoclast-specific Atx-deficient mice under physiologic condition.

Despite a significant reduction in osteoclast marker expression *in vivo* model (**Figures 3C, D**), there was no differential expression of osteoclast markers in culture model (**Figure 4C**). We think that extensive level of RANKL in culture model (100 ng/ml) might have masked the difference. It has been well established to use RANKL at a range of 50–100 ng/ml for several days in order to differentiate macrophages into osteoclasts. However, it does not reflect serum RANKL level of patients. According to a clinical study (42), serum RANKL in patients with rheumatoid arthritis was 247.92 ±124.11 pg/ml (n = 58). Additionally, the expression patterns of osteoclast markers in **Figure 4C** do not correspond to the osteoclast numbers in **Figures 4A, B**. When assessing the differentiation of osteoclasts, their morphology is crucial. Mature osteoclasts are multinucleated giant cells. In **Figure 4A**, we can see a lot of TRAP-positive premature osteoclasts. Although they were positive for TRAP staining, we do not consider them all to be osteoclasts.

Isolating lipid raft is challenging and requires careful validation with positive (e.g., flotillin, caveolin-1) and negative (e.g., transferrin receptor) controls. The methods depend on preparation factors such as detergent type, concentration, incubation duration, which should align with the tissue type studied (43) However, the expression of flotillin-1, a raft marker, is detected only in the insoluble fraction, indicating that the separation was somewhat successful (**Figure 5C**). Nevertheless, we believe our claim remains valid for two reasons. First, our main point is that the distribution of RANK, previously reported to localize primarily in lipid rafts (27), becomes dysregulated when Atx is inhibited. In our culture model, RANK was predominantly found in non-raft fractions due to inhibition of Atx. This observation is independent of the purity of the raft fractions, as our interpretation is based on the non-raft fractions, which, as shown in **Figure 5C**, did not show a flotillin-1 signal. Second, another point supporting our claim is that the raft fraction in the Atx inhibitor-treated group had less flotillin-1 than the raft fraction in the vehicle-treated group, suggesting relative impurity in the former. Despite this impurity, RANK levels were similar to those in the vehicle-treated group. Given the localization of RANK in lipid rafts, its expression should have decreased if the raft fraction was contaminated with the non-raft fraction. The fact that contamination did not reduce RANK expression suggests that RANK is present in the non-raft fraction, which we interpret as dysregulation of RANK.

Although it has been confirmed that ATX deficiency disrupts lipid rafts, it is challenging to elucidate how ATX affects lipid raft integrity. Cholesterol and its regulatory mechanisms are possible targets for maintaining ATX-mediated lipid raft homeostasis. As mentioned above, cholesterol and sphingolipids are the principal components of lipid rafts (44). Methyl-β-cyclodextrin, a well-known lipid raft-disrupting agent, alters lipid raft integrity by removing cholesterol from the cell membrane (45). This suggests that the cholesterol composition is essential for the maintenance of lipid raft integrity. Moreover, ATX has an affinity for not only LPC but also steroids and cholesterols (46). This affinity may contribute to the maintenance of the lipid raft integrity. Taken together, it can be assumed that ATX deficiency affects the dysregulation of homeostasis in lipid rafts and in the cellular membrane by modulating lipid localization and stability. Further experiments are required to confirm this assumption.

This study had some limitations that require further investigation. The finding that ATX deficiency disrupted lipid rafts is not closely linked to the anti-inflammatory and anti-osteoclastogenic effects of ATX deficiency. Owing to the lack of linkage between lipid raft disruption, arthritis, and osteoclastogenesis, it is possible that the observed anti-inflammatory and anti-osteoclastogenic effects were caused by the dysregulation of other signaling molecules, such as LPA. As many studies have reported the pro-inflammatory effects of LPA, its role should not be excluded. In addition, our previous study confirmed that LPA levels decreased in macrophages from myeloid-specific ATX-deficient mice (18). Thus, it is possible that LPA is responsible for the anti-inflammatory and anti-osteoclastogenic effects identified in this study. Therefore, it would be beneficial to investigate the effects of ATX inhibition on LPC depletion or LPA receptor inhibitory status. Furthermore, it would be promising to compare the effects of other lipid raft-disrupting agents with those of ATX deficiency in arthritis and osteoclastogenesis. Another future research direction is a close examination of the ruffled borders of osteoclasts. Ruffled border formation is important for effective bone resorption by osteoclasts (47). This structure provides excess surface area for the secretion of proteolytic enzymes into the resorption area (48). The disruption of lipid rafts can disrupt membrane integrity in osteoclasts (26). This can negatively modulate ruffled border formation in osteoclasts. In addition to osteoclastogenesis, differentiated osteoclasts do not exert their activity because of the malformation of the ruffled border. We confirmed bone resorption activity *in vitro* using a bone resorption assay with bovine bone slices (**Supplementary Figure S1**). There was less resorbed area in Atx^ΔME/ΔME^ cells compared to that in Atx^+/+^ cells. However, this did not directly imply less bone resorption activity in Atx^ΔME/ΔME^ cells because there was less osteoclast differentiation in Atx^ΔME/ΔME^ BMMs compared to that in Atx^+/+^ BMMs, which can lead to less resorbed area.

Though our mouse model is a myeloid-specific knockout model, circulatory ATX levels could potentially be affected. However, we believe that even if the circulating ATX has been affected, the extent should be negligible. Nonetheless, it would be interesting to explore the potential pathway regulated by ATX in the near future. ATX is responsible for the majority of extracellular LPA production. In our previous study, we found that total LPA and certain molecular species of LPA were reduced in ATX-cKO macrophages compared to WT control (19). One recent study suggested that LPA stimulation can induce the development of macrophages through induction of Akt/mTOR signaling pathway and PPARγ activation in mice and humans, indicating that LPA is capable of regulating immune defense mechanisms (49) Accordingly, it is possible that reduced LPA levels in the Atx-deficient mice may dampen the macrophage development and relevant inflammatory responses like rheumatoid arthritis, leading to compromised immunity.

Furthermore, ATX can bind to lymphocytes, enhancing their migration and promoting entry into lymphoid organs from the blood (50). Given that CIA is a model for autoimmune RA, it can influence both innate and adaptive immune responses. During the development of CIA, various cell types from the myeloid lineage, such as neutrophils, macrophages, monocytes, and Treg lymphocytes, can be affected. Thus, we aimed to investigate the effects of Atx on these cells within the joint. We analyzed CD31 as a T cell marker and F4/80 as a macrophage marker in the ankle tissue of Atx^+/+^ and Atx^ΔME/ΔME^ mice. Under CIA conditions, we observed a slight decrease in the number of CD31 and F4/80 positive cells in the ankle cartilage of Atx^ΔME/ΔME^ mice compared to Atx^+/+^ mice (**Supplementary Figure S2**). CD31 (also known as PECAM-1) plays a role in interactions between immune cells and is involved in both the early and late stages of T cell differentiation (51–53). This suggests a potential link between the reduced T cell-related immune response and the alleviation of arthritis symptoms in Atx^ΔME/ΔME^ mice. Additionally, previous studies have reported that macrophages in Atx^ΔME/ΔME^ mice exhibit impaired functionality (18). The reduced inflammatory response due to defective macrophages in Atx^ΔME/ΔME^ mice may contribute to the pathological features observed in the CIA model. However, we believe that a decrease in osteoclastogenesis alone is not sufficient to fully explain the reduced severity of arthritis observed in Atx^ΔME/ΔME^ mice.

We also investigated the role of LPS in osteoclastogenesis in relation to the ATX-deficiency-induced dysregulation of TLR4 signaling. LPS is a representative pro-inflammatory molecule as it activates the TLR4 signaling pathway in immune cells (54). Based on the TLR4-mediated inflammatory pathway, it may be natural that LPS facilitates osteoclastogenesis and inflammatory responses in BMMs. However, the simultaneous treatment with LPS and RANKL inhibited osteoclastogenesis. Although the role of LPS in osteoclast differentiation has been debated, this inhibitory effect of LPS was consistent with a previous finding (24). In addition, LPS inhibits osteoclastogenesis, even during ATX deficiency. Considering that our previous study confirmed that TLR4 signaling is dysregulated in ATX-deficient macrophages, it was assumed that LPS may inhibit osteoclastogenesis through ATX/TLR4-independent pathways (18). Further studies should investigate whether a downstream molecule of the TLR4 signaling pathway directly inhibits RANKL-induced osteoclastogenesis or whether a cytokine induced by LPS modulates osteoclastogenesis in an autocrine manner.

**Figure 6:**
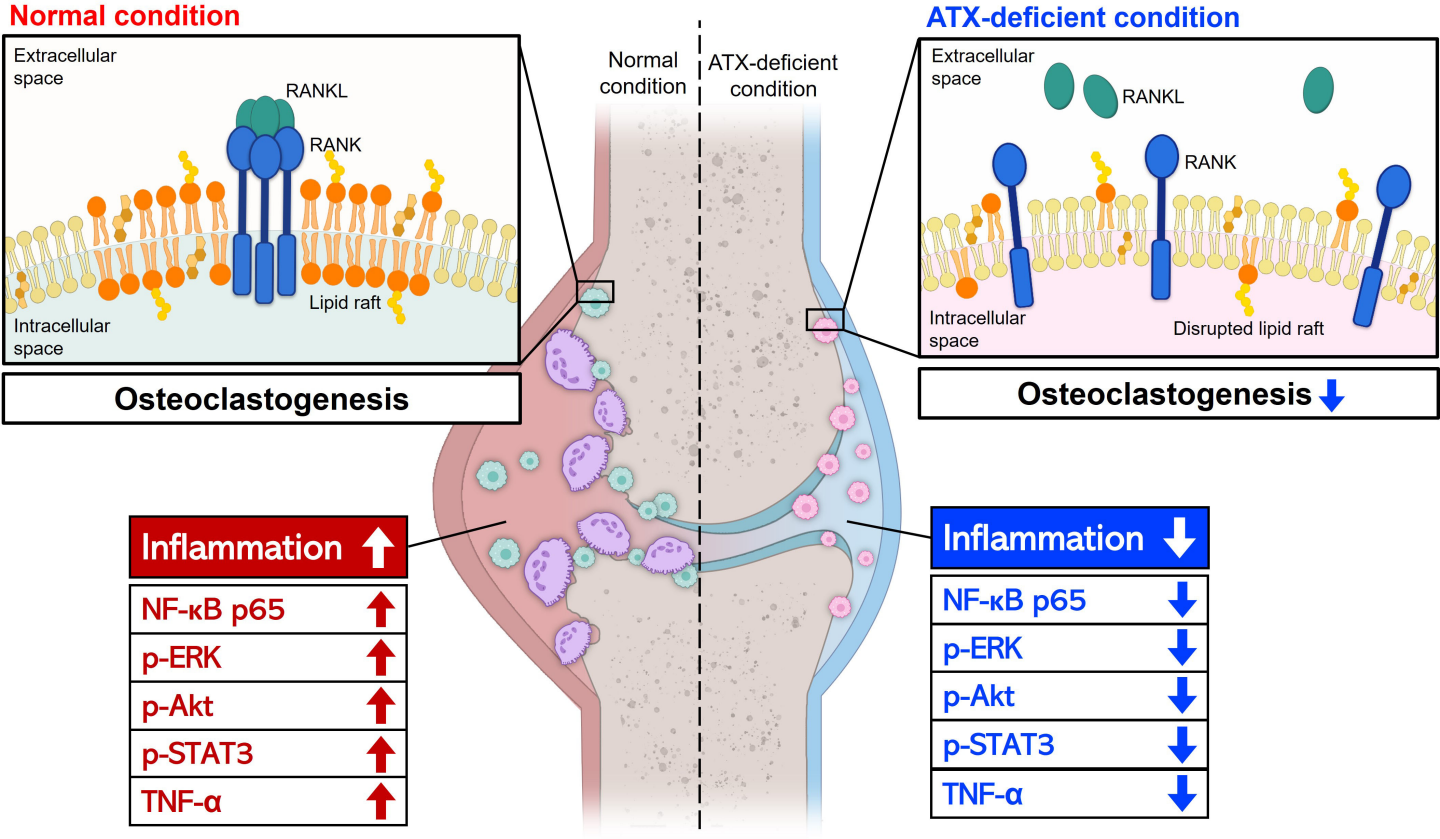
A schematic representation of the effects of ATX deficiency on joint inflammation and osteoclast differentiation. ATX deficiency suppressed joint inflammation and osteoclast differentiation, thereby hindering bone and cartilage destruction. Anti-inflammatory effects of ATX deficiency were mediated by reduced p-STAT3, p-ERK, p-Akt, NF-κB, and TNF-α levels. Lipid raft disruption and abnormal RANK distribution may contribute to the ATX deficiency-mediated anti-osteoclastogenic effects. LPS inhibited osteoclast differentiation and the inhibitory effect was independent of ATX. Arrow-headed lines represent differentiation, promotion, or progression. Bar-headed lines indicate blockade. ATX, autotaxin; STAT3, signal transducer and activator of transcription 3; ERK, extracellular signal-regulated kinase; Akt, Protein kinase B; NF-κB, nuclear factor kappa B; TNF-α, tumor necrosis factor alpha; RANK, receptor activator of nuclear factor kappa B; LPS, lipopolysaccharide.

The present study showed that ATX deficiency suppresses inflammation of the joint and differentiation of osteoclasts, which mediate bone and cartilage destruction (**Figure 6**). Because RA is a progressive disease, it is important to slow down or block bone destruction to improve the prognosis. ATX inhibition can hinder the progression of RA by suppressing osteoclast differentiation and alleviating inflammatory symptoms, such as joint pain and swelling. In conclusion, ATX inhibition may be a promising strategy for the development of new DMARDs.

## Data Availability

The datasets presented in this study can be found in online repositories. The names of the repository/repositories and accession number(s) can be found in the article/**Supplementary Material**.
