# Supplementary material for: Myeloid-specific deletion of autotaxin inhibits rheumatoid arthritis and osteoclastogenesis
Source: Front Immunol. 2024 Dec 18;15:1481699. doi: 10.3389/fimmu.2024.1481699 (PMC11688342; doi:10.3389/fimmu.2024.1481699)
Supplement: Supplementary file 1 [file DataSheet1.docx]

Supplementary Material

# Supplementary Figure


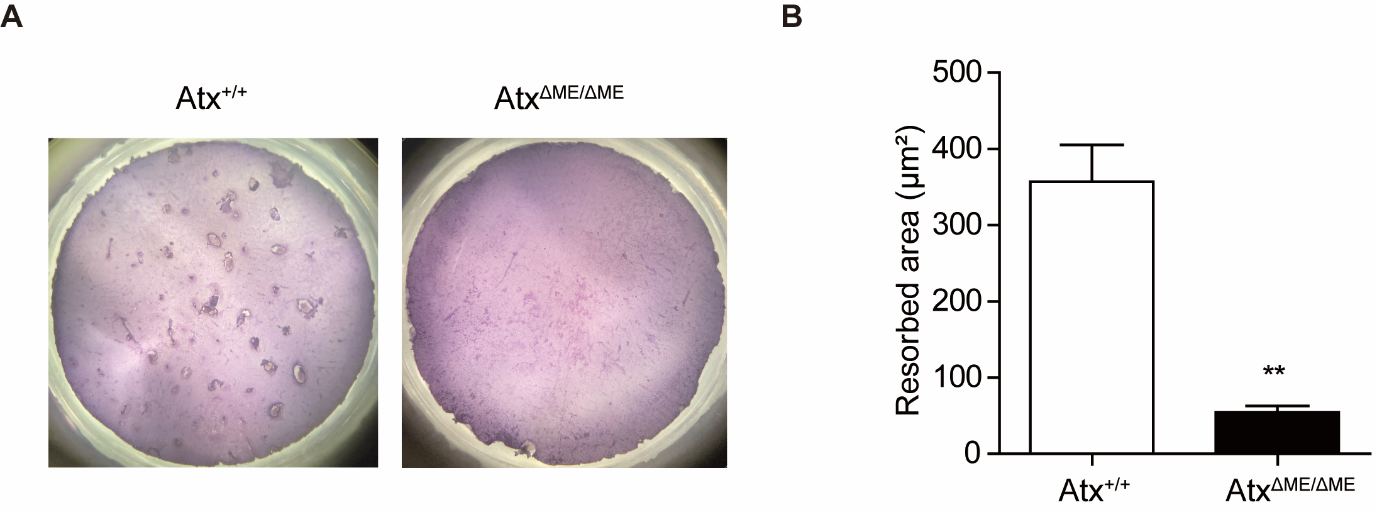


**Supplementary Figure S1.** Atx^ΔME/ΔME^ cells showed less bone resorption activity compared to Atx^+/+^ cells. (A) Bone resorption activity of differentiated osteoclasts was analyzed by bone resorption assay. Bone marrow-derived macrophages were cultured and differentiated into osteoclasts on the bovine bone slices. (B) Resorbed area was measured with Image J software. **p < 0.01.


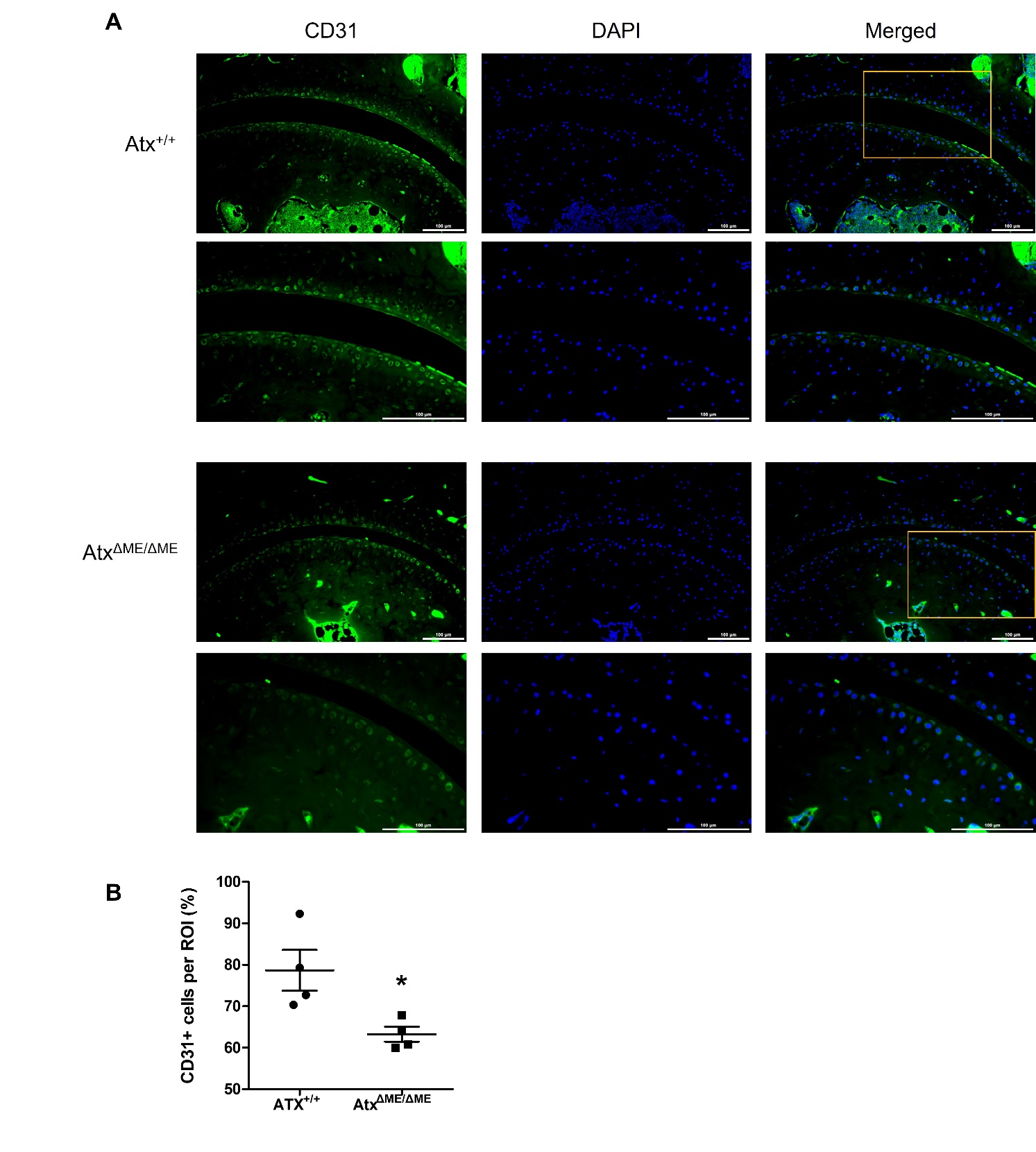


#
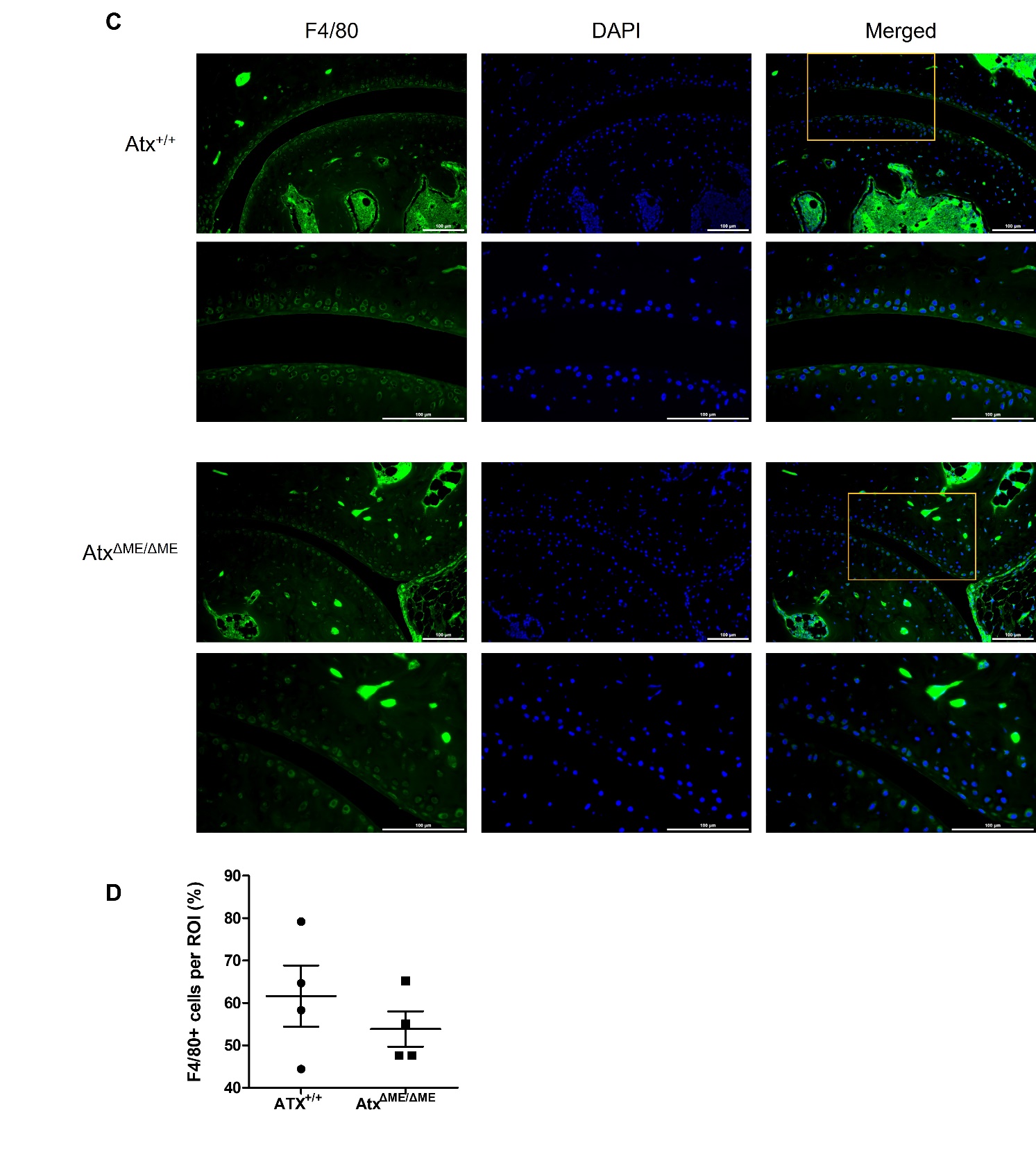


**Supplementary Figure S2.** Myeloid-specific deletion of Atx reduced the number of macrophages in the cartilage in a CIA mouse model. (A and C) Ankle tissue from Atx^+/+^ and Atx^ΔME/ΔME^ was fixed using formalin, decalcified using EDTA solution, embedded in paraffin, and then incubated with anti-CD31 and anti-F4/80 antibodies. (B and D) The cells in the lining of the cartilage were counted. The ratio of CD31+ cells to CD31- cells and F4/80+ cells to F4/80- cells per region of interest (ROI) was plotted. The yellow squares indicate the areas of observation in the lower panels. *p < 0.05.
